# Supplementary figures and images for: In Vitro Infection Model Using A6 Cells Sets the Stage for Host–Batrachochytrium salamandrivorans Exploration
Source: J Fungi (Basel). 2025 Feb 18;11(2):156. doi: 10.3390/jof11020156 (PMC11856035; doi:10.3390/jof11020156)

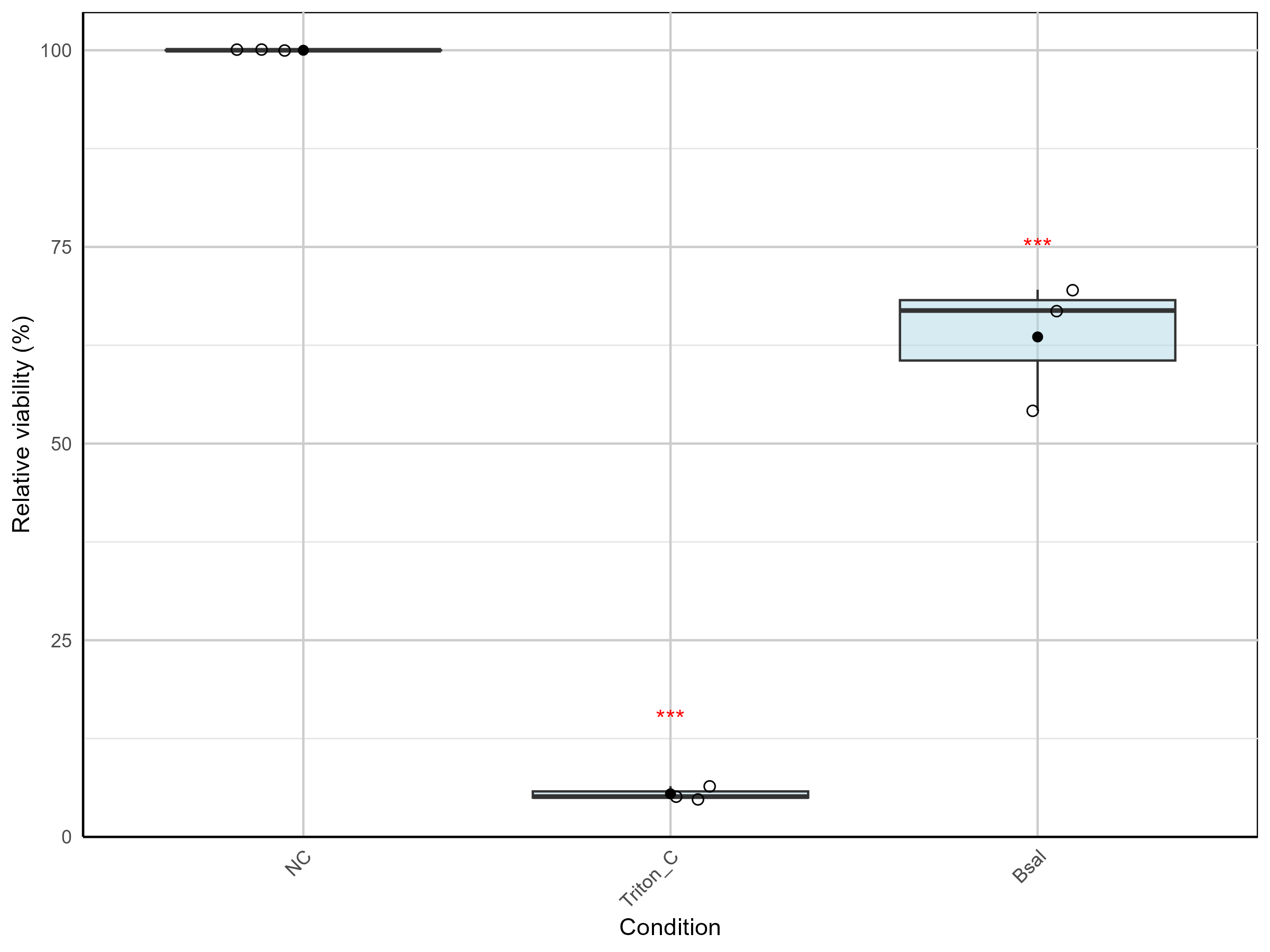

Supplement: Supplementary file 1 [file jof-11-00156-s001.zip › Figure S1.tiff]

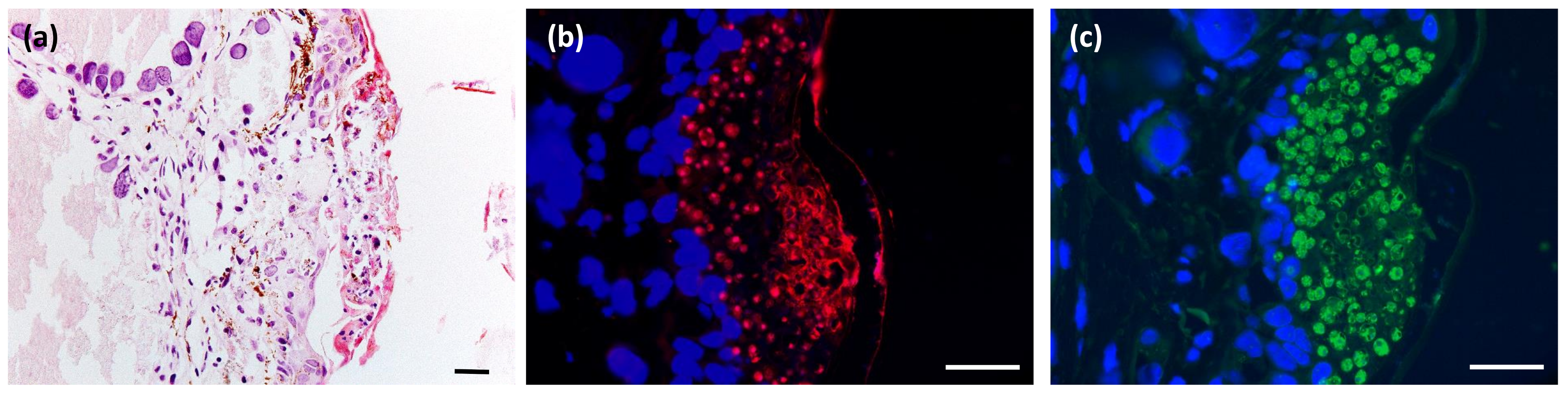

Supplement: Supplementary file 1 [file jof-11-00156-s001.zip › Figure S2.tiff]

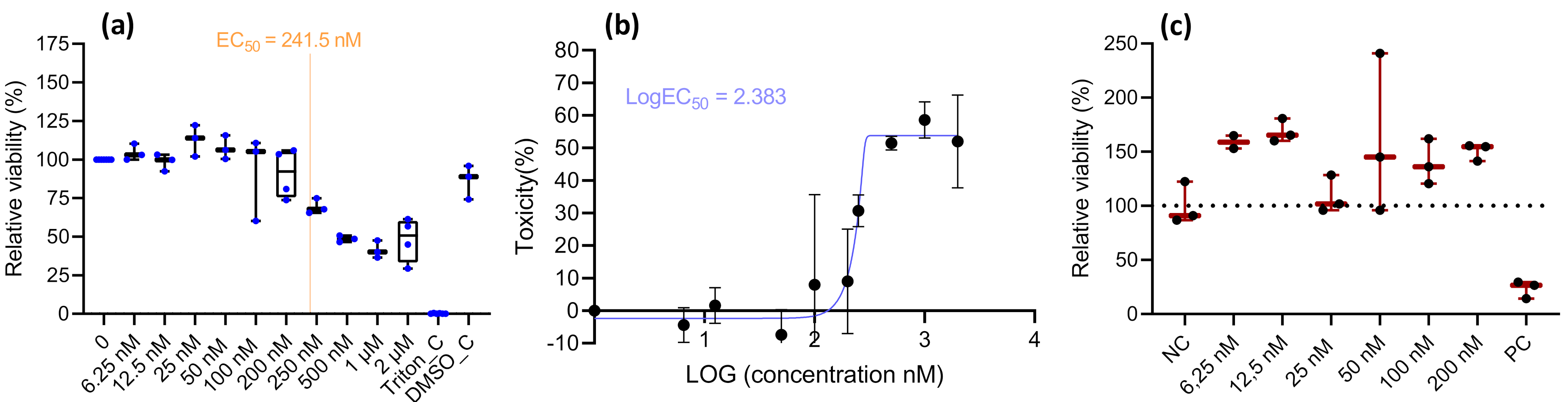

Supplement: Supplementary file 1 [file jof-11-00156-s001.zip › Figure S3.tiff]

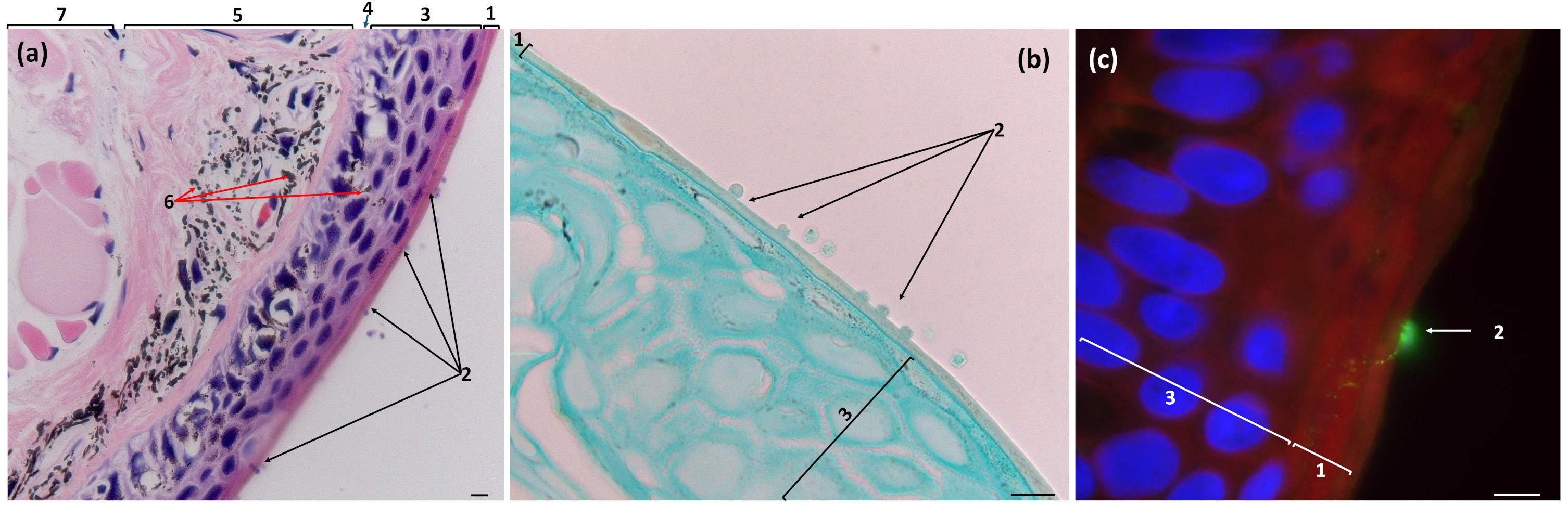

Supplement: Supplementary file 1 [file jof-11-00156-s001.zip › Figure S4.tiff]
